# Estimating regional and national cancer incidence in Uganda: a retrospective population-based study, 2013–2017

**DOI:** 10.1186/s12885-024-12543-9

**Published:** 2024-07-02

**Authors:** Annet Nakaganda, Angela Spencer, Collins Mpamani, Cissy Nassolo, Sarah Nambooze, Henry Wabinga, Isla Gemmell, Andrew Jones, Jackson Orem, Arpana Verma

**Affiliations:** 1 Uganda Cancer Institute, Kampala, Uganda; 2 University of Manchester, Manchester Academic Health Sciences Centre , Manchester, UK; 3 Kampala Cancer Registry, Kampala, Uganda; 4 Makerere University College of Health Sciences, Kampala, Uganda

**Keywords:** Estimating, Regional, National, Cancer, Incidence, Surveillance

## Abstract

**Background:**

Cancer is becoming a major health problem in Uganda. Cancer control requires accurate estimates of the cancer burden for planning and monitoring of the cancer control strategies. However, cancer estimates and trends for Uganda are mainly based on one population-based cancer registry (PBCR), located in Kampala, the capital city, due to a lack of PBCRs in other regions. This study aimed at estimating cancer incidence among the geographical regions and providing national estimates of cancer incidence in Uganda.

**Methods:**

A retrospective study, using a catchment population approach, was conducted from June 2019 to February 2020. The study registered all newly diagnosed cancer cases, in the period of 2013 to 2017, among three geographical regions: Central, Western and Eastern regions. Utilizing regions as strata, stratified random sampling was used to select the study populations. Cases were coded according to the International Classification of Diseases for Oncology (ICD-0-03). Data was analysed using CanReg5 and Microsoft Excel.

**Results:**

11598 cases (5157 males and 6441 females) were recorded. The overall national age-standardized incidence rates (ASIR) were 82.9 and 87.4 per 100,000 people in males and females respectively. The regional ASIRs were: 125.4 per 100,000 in males and 134.6 per 100,000 in females in central region; 58.2 per 100,000 in males and 56.5 per 100,000 in females in Western region; and 46.5 per 100,000 in males and 53.7 per 100,000 in females in Eastern region. Overall, the most common cancers in males over the study period were cancers of the prostate, oesophagus, Kaposi’s sarcoma, stomach and liver. In females, the most frequent cancers were: cervix, breast, oesophagus, Kaposi’s sarcoma and stomach.

**Conclusion:**

The overall cancer incidence rates from this study are different from the documented national estimates for Uganda. This emphasises the need to enhance the current methodologies for describing the country’s cancer burden. Studies like this one are critical in enhancing the cancer surveillance system by estimating regional and national cancer incidence and allowing for the planning and monitoring of evidence-based cancer control strategies at all levels.

**Supplementary Information:**

The online version contains supplementary material available at 10.1186/s12885-024-12543-9.

## Introduction

Cancer is among the leading causes of death worldwide [1–3]. Globally, 19.9 million new cancer cases are registered annualy, resulting in about 9.7 million cancer deaths [1]. The global incidence of cancer is rapidly increasing and is projected to rise to 28.4 million new cases in 2040 [2–4].

Cancer incidence estimates suggest an increase in Uganda from 27,410 new cases in 2012 to 35,968 cases in 2022. Similarly, cancer related deaths have increased from 17,120 in 2012 to 24,629 in 2022 [1, 5, 6]. The overall estimated age-standardized incidence rates for Uganda among males and females are 156.2 and 157.7 per 100,000 respectively. Cancer surveillance data in Uganda, specifically cancer incidence trends, is mainly based on one Population-based cancer registry (PBCR), the Kampala Cancer Registry (KCR). KCR was established in 1954 to obtain information on cancer occurrence in Kyaddondo county, located in central region [82]. Currently, KCR covers approximately 3.5 million people [7, 8]. Another upcoming population based registry is Gulu cancer registry, established in 2013 in Northern Uganda (about 60 years after the first registry) [83]. Gulu Cancer registry covers approximately 1 million people and started supllimenting KCR data to estimate the national cancer incidence in 2018 [9]. Taken together, these two cancer registries cover approximately 4.5 million people; about 9.6% of the Ugandan population, that is currently estimated at 46.5 million people [8, 10–12].

While the the cancer burden estimates for Uganda (based on Kampala and Gulu Cancer registry) are generated using robust methods by GLOBOCAN, these are based on the country reported data. The data from Uganda may be not be representative of the whole country because the current PBCRs cover less than 10% of the total population, are urban-based yet 80% of the population is rural and are from only two regions (central and northern Uganda). Also, geographical regions in Uganda are different culturally, environmentally and socioeconomically, and this may affect the burden and distribution of diseases, including cancer [7, 13].

Therefore, there is a need to estimate the cancer incidence for other geographical regions to increase our understanding of the epidemiology of cancer in Uganda and to direct the cancer control effort at local levels [14, 15]. The lack of cancer estimates in other regions of the country is due to the gap in cancer registration and lack of a clear national framework for cancer surveillance research in Uganda [16]. The inadequate infrastructure for cancer surveillance resonates with the fragile health system that lacks capacity for cancer research and control including: human resource; funding; treatment and diagnostic facilities; and an adequate medical records management system [14, 17, 18]. As a result, establishing and maintaining cancer registries, of good quality, in different regions is not yet feasible for many resources constrained countries such as Uganda [19, 20]. This has been demonstrated by the upcoming cancer registry in Northern Uganda showing that even when registries are established, it takes time before they can produce high quality cancer statistics that meet international standards [9, 16, 19, 21].

Hence, this study aimed at estimating regional and national cancer incidence, using a retrospective catchment population approach. Regional estimates may improve national estimates of the cancer burden, and enable rational planning and implementation of targeted cancer control programs. It will also allow assessment of any variations in cancer occurrence and direct future research into the causes and prevention of cancer in Uganda.

## Materials and methods

### Study design and population

A retrospective study, using a catchment population approach was conducted in Uganda from June 2019 to February 2020. This study registered all newly diagnosed cancer cases in the period of 2013 to 2017 among three geographical regions: Central, Western and Eastern regions. The study period of 2013–2017 was chosen because it was the recent five-year period at the time of conducting the the study. The study methods were first tested in a feasibility study that was conducted in 2018 in one of the Ugandan districts and proved feasible for the study objectives [22]. The study population was selected using stratified random sampling. Utilizing regions as strata, a simple random sample of five districts was drawn from each region. The five districts per region were chosen based on scientific reasons (to provide a study population that was large enough to provide robust cancer estimates for a relatively rare disease such as cancer) and other factors such as cost, practical operations and logistic issues, as well as the context and setting.

### Data sources and method of data collection

Data was collected from all health facilities within the selected districts: and from regional and national health facilities that were known to diagnose and treat cancer patients for the study districts. In addition, information from major laboratories and a cancer registry in the country was reviewed to find cancer cases that belonged to the selected districts. Data was collected by active collection, which involved the research team visiting different sources and abstracting data on paper-based Data Abstraction Forms. The Data Abstraction Form was developed based on International Agency for Research on Cancer (IARC) recommendations for cancer registration and included all the mandatory variables for estimating cancer incidence in a population [23, 24]. Variables collected were: patient names; residence address; age; sex; cancer incidence date; basis of diagnosis; primary site; histological type; behavior of the tumour; stage of disease; treatment received; date of last contact; and status of last contact.

The researchers identified cancer cases by examining general attendance registers and medical records of the health facilities across various departments, including: outpatient and in-patient departments; oncology clinics; HIV clinics; cancer screening clinics; records department; admission and discharge forms; pathology and laboratory records; autopsy reports; radiology/imaging records; radiotherapy department; and mortuary registers and death certificates.

### Eligibility criteria and case definition

All cancer cases for all age-groups that occurred in the selected districts, within the study period of 2013–2017, were included in the study. A cancer case was defined as any cancer patient diagnosed either based on clinical history; or clinical history with other investigations like x-ray, microscopic, cytology, autopsy, histology (microscopic); or immunohistostaining. This case definition is in line with the general guidelines for cancer registration set by IARC [25].

### Data analysis

Cases were coded according to the International Classification of Diseases for Oncology (ICD-0-03). Data was managed and analysed using CanReg5 and Excel. Incidence rates were age-standardized using the world standard population. Cumulative rates were also calculated over the five-year period and used to estimate the cumulative risk of developing cancer among the study populations.

### Source of the total population denominator

Population estimates to allow calculation of person-years at risk for this study were obtained from Uganda Bureau of Statistics (UBOS) population census estimates. UBOS conducted the latest national population and housing census for Uganda in 2014 and the population of the districts and regions is available by sex and 5-year age group. The 2014 population figures were used for this study and also used to calculate post-censal projections of the population size and structure for other subsequent years. The 2014 national population census estimated an annual population growth rate of 3.0%. This rate of change was applied to all age-groups to calculate estimates of the population in 2015, 2016 and 2017, assuming a constant rate of change within age-sex groups [26]. The 2013 population estimates were derived using simple linear regression trend analysis.

### Accuracy of the population denominator estimates

Uganda conducts its national census every 10 years, and always calculates the average annual growth rates for the Inter-censal period. For example, the average annual growth rate was 2.7, 2.5, 3.2 and 3.0 for 1980, 1991, 2002 and 2014 respectively. These rates are used to calculate Inter-censal and post-censal annual population estimates, assuming a constant rate of change [8]. However, the accuracy of Inter-censal and post-censal estimates may be comprised by the high rate of temporally rural-urban migration and internal displacement of people due to political reasons and other social-economic and environmental conditions [7, 23, 27, 28].

The census enumeration is carried out through face to face interviews and conducted on a de facto basis (consisting of those who slept in the villages the night before the census) [7, 28]. Special measures are always undertaken to assure quality and validity of the data including: adequate training and supervision of census staff; subdivision and mapping of the country into manageable Enumeration Areas; and use of Post Enumeration Surveys (PES) to evaluate the quality, completeness and accuracy of the census data [7]. Results from one of the census post-evaluation surveys showed that the national coverage rate was 94.4% with an omission rate of 5.6%. The national erroneous inclusion rate was 3.6% and the gross coverage error rate was 9.2%. These figures are comparable with data from other countries in the sub-Saharan region [29].

### Ethical approval

Ethical approval was obtained from Uganda Cancer Institute and University of Manchester Research Ethics Committees. Further regulatory clearance was obtained from the Uganda National Council for Science and Technology and from all the health facilities that provided data for the study.

## Results

### Study population

Fifteen districts (five districts from each region) were included in the study, providing a total study population (denominator) of 5,348,308 people. The majority of the study population and registered cancer cases were from Central region, followed by Western region and Eastern region respectively (see Table 1). Data was collected from several sources including: Cancer registry, contributing 22% of the registered cases; general hospitals, contributing 21% of the cases; National cancer center, contributing 19% of the cases; pathology laboratories, contributing 17% of the cases; Hospice centers, contributing 13% of the cases; and Health Centre IV level facilities (HCIV), which contributed only 1% of the registered cases.

**Table 1:** Study population, by region, district and recorded cancer cases

Study Population (region and districts)		^*^Total population denominator	Total cancer cases no.	^**^Cases %
**Central**		**2,568,638**	**7622**	**65.7**
	Kampala	1,507,080	5810	50.1
	Kayunga	368,062	426	3.7
	Masaka	297,004	1016	8.8
	Kyankwanzi	214,693	201	1.7
	Nakasongola	181,799	169	1.5
**Western**		**1,511,765**	**2376**	**20.5**
	Mbarara	472,629	1178	10.3
	Kamwenge	414,454	327	2.8
	Kiruhura	328,077	445	3.8
	Mitooma	183,444	344	3.0
	Buliisa	113,161	82	0.7
**Eastern**		**1,267,905**	**1600**	**13.8**
	Mbale	488,960	922	7.9
	Amuria	270,928	278	2.4
	Butalejja	244,153	207	1.8
	Bulambuli	174,508	115	1.0
	Bukwo	89,356	78	0.7
**Total**	**Overall (All regions/districts)**	**5,348,308**	**11598**	**100**

*Total population denominator: is the total number of people in the district or region according to UBOS 2014

**Cases (%) was calculated basing on the overall total cases identified

### Overall national cancer incidence rates in Uganda

**Fig. 1:**
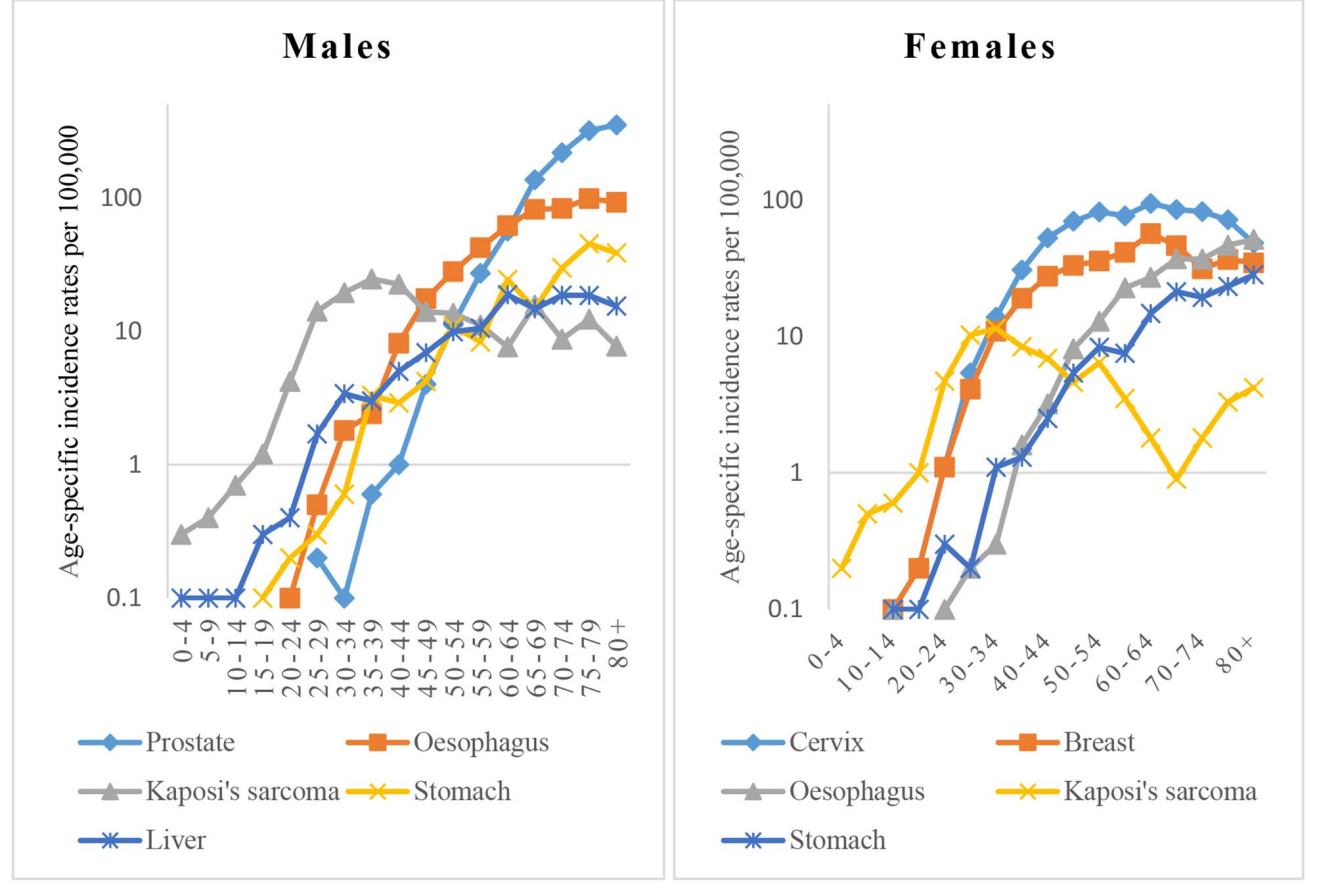
Age specific incidence rates for the most common cancers in Uganda

During the five-year study period (2013–2017), 11,598 cases (5157 males and 6441 females) were registered in the three regions of Central, Western and Eastern. In females most of the recorded cases were in the 35–54 year-age groups, while in males, most cases were aged 54 years and above, Fig. 1. About 72% (71.5%) of the cases were diagnosed morphologically. The overall age-standardized incidence rates were 82.9 per 100,000 people (SE = 1.29; 95% CI: 79.35–84.4) for males, and 87.4 per 100,000 for females (SE = 1.2; 95% CI: 84.05–88.76). These confidence intervals only slightly overlap indicating that the difference between males and females is too close to be statistically significant. In males, the most common cancers over the period were cancers of the prostate, oesophagus, Kaposi’s sarcoma, stomach and liver. In females, the most frequent cancers over the study period were cancers of the cervix, breast, oesophagus, Kaposi’s sarcoma and stomach (see Appendix 1 for details).

Although prostate and cervix are the commonest cancers overall, different cancers predominate in different age-groups. According to age-specific incidence curves (Fig. 1), the predominant incident cancer in the younger age groups (0–44 years among males, and 0–34 among females), is Kaposi’s sarcoma. In males, cancer of the oesophagus is the commonest cancer in the middle age groups of 45–64 years, while prostate is commonest in 65-year and above age-groups. In women, cancer of the cervix is the commonest cancer among 35 years and above age-groups.

The risk (using cumulative rates) of developing any form of cancer was 9% for both males and females, implying that 1 in 11 people would develop cancer before the age of 75 years (see Appendix 1).

### Cancer incidence in central region

7,727 cases were registered in Central region (3,426 males and 4,301 females). 80% **(**80%) of the cases were diagnosed morphologically and 0.4% cases were from death certificates only. The mean age at diagnosis, for both sexes combined, was 46.4 years. The Age-standardised cancer incidence rates (ASIRs), for all cancers combined, in Central region were 125.4 per 100,000 in males (SE = 2.54; 95%: 118.7-128.67) and 134.6 per 100,000 in females (SE = 2.39; 95% CI: 128.3-137.67 (see Appendix 2). The top five cancers diagnosed in males, over the study period, are prostate, oesophagus, Kaposi’s sarcoma, liver and stomach. In females, the most common malignancies are cervix uteri followed by breast, oesophagus, Kaposi’s sarcoma and liver. Although cervix and prostate are the commonest cancers overall, different age groups are heavily affected by different cancers in both sexes.

**Fig. 2:**
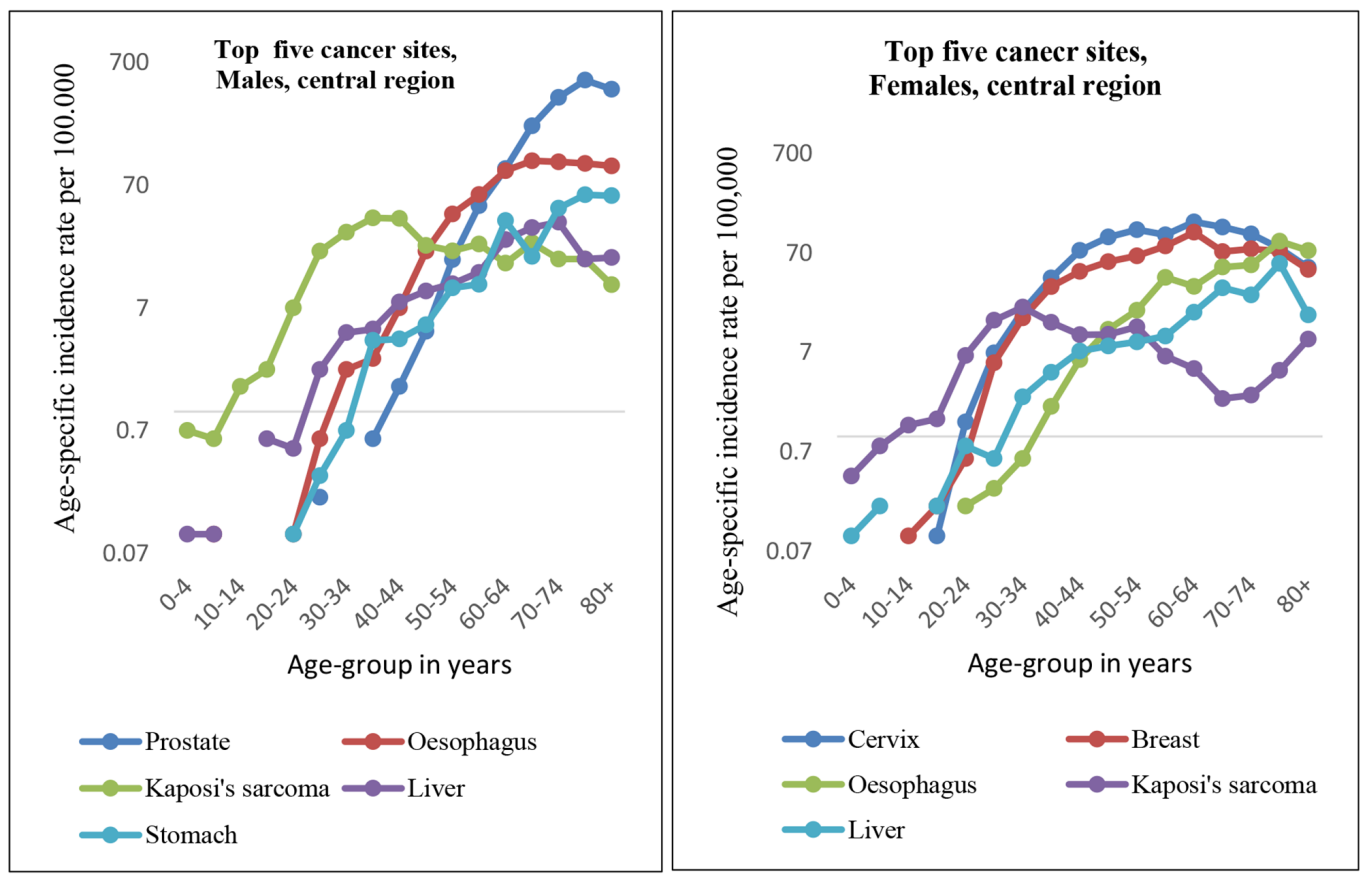
Top five cancers, Central region, by age group and sex

Among males, Kaposi’s sarcoma is the commonest cancer in the 20-44-year age groups; oesophagus in the 45-64-year age groups; and prostate cancer commonest among 65-years and above age groups. Similarly, Kaposi’s sarcoma is the commonest cancer among the 15–34 year females and cervix commonest after the age of thirty-five years (see Fig. 2).

The risk (using cumulative rates in Appendix 2) of developing any form of cancer was 15% for both males and females, implying that 1 in 7 people, in Central region, would develop the disease before the age of 75 years.

### Cancer incidence in western region

2,413 cases were registered in Western region: 1,131 men and 1,282 women. 66% of the cases were diagnosed morphologically and 0.1% were death certificate only cases. The mean age at diagnosis was 50.3 years. In women, most cancers were diagnosed in the 40 to 64- year age groups, while in men most cases were from the 65-year and above age groups. The Age-Standardised cancer incidence rates (ASIR), for all cancers combined, in Western region were 58.2 per 100,000 in males (SE = 1.96; 95% CI: 54.42–61.93) and 56.5 per 100,000 in females (SE = 1.71;95% CI: 51.96–58.66), Appendix 3. Overall, the most commonly diagnosed cancer in males was prostate, followed by oesophagus, stomach, Kaposi’s sarcoma and liver. In females, the most common malignancy was cervix uteri followed by breast, stomach, oesophagus, and ovary. Unlike in Central region, Kaposi’s sarcoma and liver cancers are not among the top five cancers for females in Western region; instead they are replaced by stomach cancer (3rd commonest cancer) and ovary (5th commonest).

**Fig. 3:**
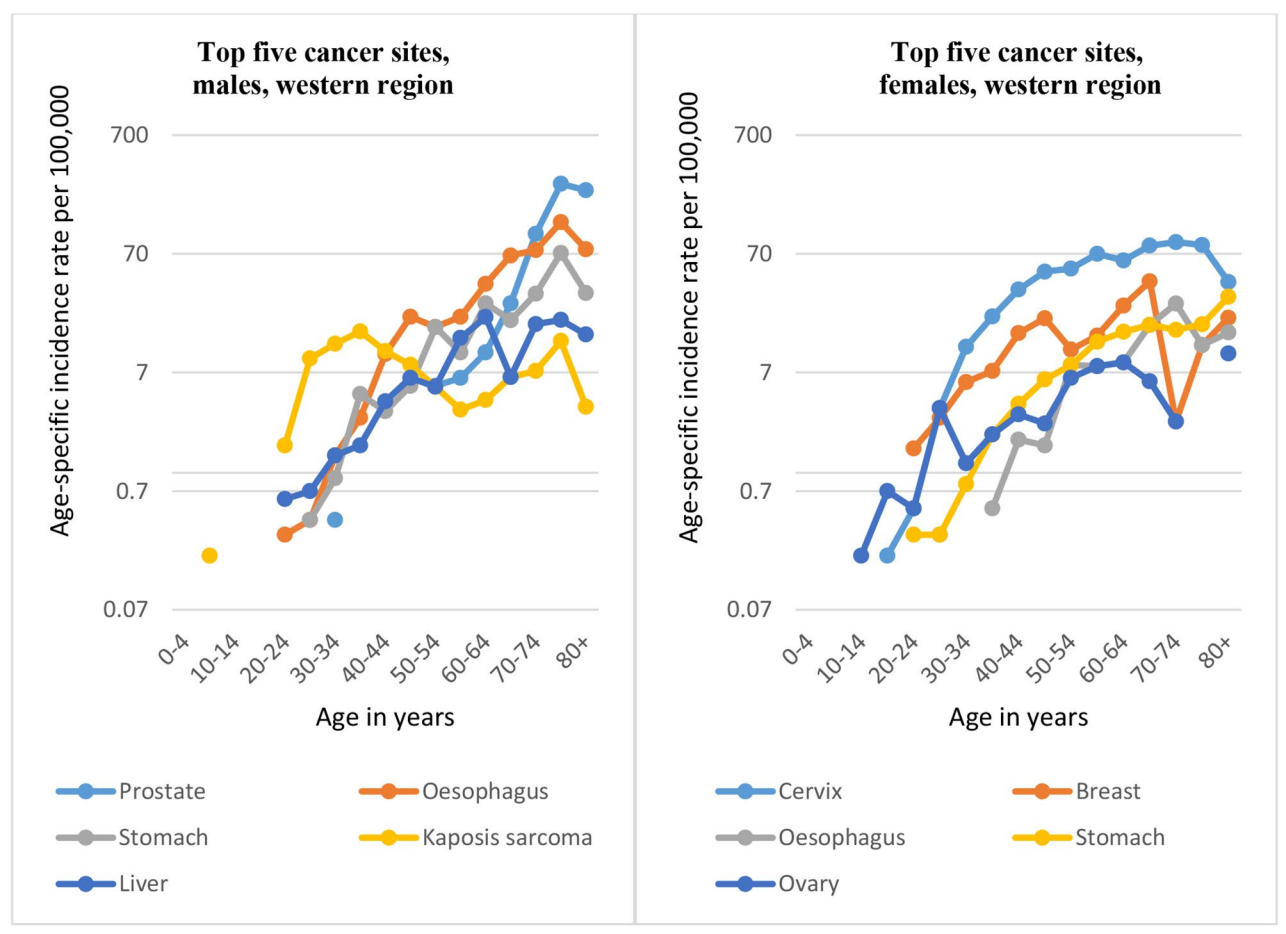
Top five cancers in Western region by age group, 2013–2017

However, as in Central region, Kaposi’s sarcoma is the commonest cancer in Western region in males aged 20–44 years, oesophagus for the 45–74 age groups and prostate becomes commonest after the age of 75 years. Among females, ovary is the commonest cancer for the 10-24-year age groups, after which cancer of the cervix seems to be the commonest cancer for all female age groups in this region, Fig. 3. The cumulative risk (cumulative rate) of developing any form of cancer in Western region is 6% for both men and women, implying that 1 in 17 people will develop cancer before 75 years of age.

### Cancer incidence in eastern region

1,623 cases were registered in Eastern region: 666 men and 957 women. 44% of the cases were diagnosed morphologically. As in other regions, prostate is the most commonly diagnosed malignancy in men (with 162 cases), followed by oesophagus (130 cases). Similarly, cervix is the most commonly diagnosed malignancy in women, with 320 cases, followed by breast (120 cases). The mean age at diagnosis, both sexes combined, was 50.6 years. Among women, most of the cases were diagnosed in the 44-64-year age groups, while in men most cases were diagnosed in the 55-74-year age groups. The Age-standardised incidence rates (ASIRs) in eastern region, for all cancers combined, were 46.5 per 100,000 in males (SE = 1.92; 95% CI: 41.76–49.29); and 53.7 per 100,000 in females (SE = 1.81; 95% CI 48.71–55.82). Overall, the most commonly diagnosed cancer among males in Eastern region was prostate, followed by oesophagus, Kaposi’s sarcoma, liver and colon. In females, the most common malignancy was cervix uteri followed by breast, oesophagus, stomach and liver cancers (see Appendix 4).

**Fig. 4:**
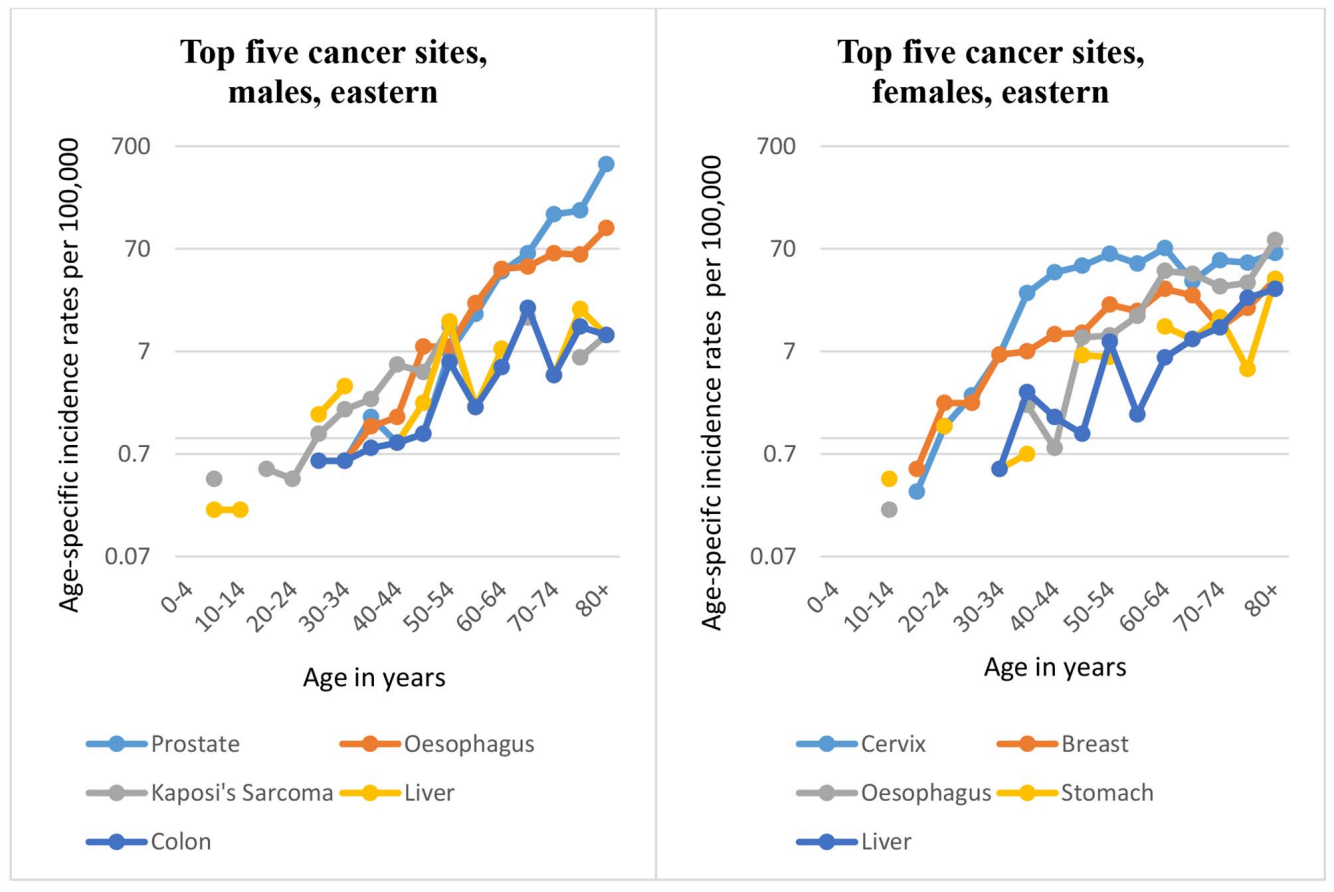
Top five cancers in Eastern region by age group

Considering the age-specific rates, as in other regions, Kaposi’s sarcoma is the commonest cancer among males aged 20-44-years; oesophagus for the 45–64 age-groups; and prostate commonest after the age of 65 years. Among females, breast seems to be the commonest cancer for the 15-34-year age groups, after which cancer of the cervix becomes the commonest cancer for all female age groups in this region (see Fig. 4). The cumulative risk of developing cancer in Eastern region is 5% in men and 6% in women, implying that one in twenty men and one in seventeen women will develop the disease before the age of 75 years.

## Discussion

This study set out to determine cancer incidence among geographical regions and to estimate the national cancer incidence in Uganda. While establishing regional PBCRs may be more ideal, the challenges to establishing and maintaining such registries are still enormous in resource limited countries like Uganda. Our successful use of a retrospective population-based approach to establish cancer incidence rates, among the regions and populations of the country, is an example of alternative actions that can be pursued to improve estimates nationally and ensure that local quality data and timely evidence are available for guiding targeted cancer control strategies and programmes in the country.

The overall age-standardised incidence rates observed in this study, all ages and all cancers combined (82.9 per 100,000 people for males and 87.4 per 100,000 for females), are far below those observed by the Kampala cancer registry, a population-based cancer registry located in the capital city (2011–2013 rates of 162.1 and 182.0 per 100,000 among males and females respectively) [8]. The rates are also lower than the 2018 GLOBOCAN estimates for Uganda (overall ASIRs of 151.7 per 100,000 among males and 154.5 per 100,000 among females). The low age-standardised rates in our study are due to the better capture of non-urban populations in the study sample compared to the existing registry. Several factors could explain this observation including: underutilisation of medical facilities by rural populations; underdiagnoses of cancer cases by rural health facilities; treatment based migration to urban areas; underreporting of rural cancer cases by our study; or there may be a true difference in the risk of cancer and the magnitude of exposure to cancer risk factors between urban and rural populations in Uganda [30, 31]. Overall, these findings offer some evidence that a country like Uganda, where 80% of the population lives in rural areas, may not benefit from utilising estimates from urban population-based cancer registries only [7, 14, 17, 32].

Similar urban and rural differences in cancer incidence rates have been documented in many countries including Gambia, India, Ghana, Kenya, and Egypt among others [31–35]. Although the importance of each of the factors contributing to these differences is difficult to judge, these repeated observations call for action: further work and studies, which take these factors into account, will need to be undertaken to adequately investigate why lower incidence rates are registered in rural areas. This will assess the contribution of each factor and objectively differentiate and quantify the underestimation due to systematic factors and underestimation due to the actual differences in cancer risk and exposure to cancer risk factors. Otherwise, a general assumption that very low rates in rural settings imply under reporting of cases due to inadequate cancer infrastructure may obscure the assessment of true and actual differences and prevent understanding of the aetiology of cancer in African populations. In addition, it will mask the appreciation of the differences in cancer risk and magnitude of exposure to cancer risk factors that would ultimately direct targeted cancer control interventions among different populations of the country.

Another important finding is the similarity between the types of cancers observed in this study with those previously described in Uganda and Sub-Saharan Africa [8, 21, 33]. The commonest types of cancers registered in this study, among all the regions, were: cervix, prostate, breast, oesophagus, liver, stomach, Kaposi’s sarcoma, ovary and colorectal. These results and the overall high proportion of female cases are consistent with those described previously by the Kampala Cancer registry and in other parts of Sub-Saharan Africa [31, 33, 36]. This implies that even when population-based cancer registries are eventually established among different regions in Uganda, studies like this one are equally important to supplement and validate the data from PBCRs [30, 37]. Although comparison of PBCR data with independent cohort studies or community surveys is widely used and is the recommended method to investigate the completeness of registration of cancers in a registry, for various reasons, this has never been considered for Uganda [38, 39]. Such studies, therefore, can be a great resource for enhancing cancer surveillance research in the country.

As far as data quality is concerned, the findings suggest that the data generated in this study are reasonably comparable and accurate. In terms of comparability, the study used the IARC international guidelines and recommendations for collecting and abstracting the data. Cases were also coded and classified according to the 3rd edition of International Classification of Diseases for Oncology (ICD-0-03) and incident dates and multiple primaries defined and determined according to the European Net-work of Cancer Registries algorithm [40, 41]. In addition, the overall proportion of morphologically verified (MV%) cases was 72%. This meets and exceeds the SSA standards of 61%, although this differed among the three regions [42]. The differences among the regions indicate that Central region MV% of 80% is comparable to standards in high income countries, while Eastern region MV% of 44% is less than SSA standards. This shows huge inequalities in cancer services within the country, as Eastern region seems to rely more on clinical cancer diagnosis, compared to other regions [33, 40]. Similarly, the proportion of cancers identified through death certificates from hospital mortuary was 0.3% and is in line with international standards, although this could be due to the low autopsy rate and inadequate vital statistics data in the country. There was no missing information for mandatory variables like age, sex, topography, morphology, address, incident date and basis of diagnosis. The proportion of ill-defined sites and primary site uncertain (combined), was 2.1% for women and 2.5% for men; and more prevalent in older age-groups.

Although the completeness of data has to be improved in Uganda (according to international quality indicators related to health-care infrastructure), this study provides a first step towards assessment of the regional and national cancer incidence; by rigorously attempting to document all the cancer cases diagnosed and treated in health facilities that serve the studied regions [19]. In the absence of any form of population-based cancer data among these regions, this data provides a unique insight into the cancer patterns in these regions and such periodic studies may be the only mechanism to monitor the disease in these regions now. In addition, such regional studies are needed now for generating local and timely evidence that can be easily comprehended by the policy makers and government officials. It is much easier to engage policy makers and politicians in cancer control when presenting real data from their respective settings and regions than presenting modelled estimates [18, 35]. Locally generated cancer data, even when not adhering to all international standards, play a big role in pointing-out strengths and weakness of the whole healthcare system and may assist in quantifying the magnitude of the regional problems, providing evidence for the needed improvements in cancer care [16, 43]. Hence, the results of this study will demonstrate to policy makers how important their support is in improving the quality of cancer data in Uganda and could allow for policy development, appropriate planning and placement of cancer diagnostic and treatment facilities in different parts of the country. In addition, these studies, if done periodically, can be used to assess the implementation of cancer prevention strategies and provide an understanding of the long-term effects of cancer control interventions in different populations.

### Limitations and strengths of the study

The most important limitation of the study was the inability to assess the completeness of the data using quantitative methods like independent case ascertainment. We may also have underestimated the incidence in rural populations due to the factors inherent in rural health infrastructures for cancer diagnosis and treatment. Many patients in rural regions may lack access to cancer treatment services and hence are never diagnosed. However, since this is the first attempt to capture data from other regions, including mostly rural districts, it is a very great addition to the important work on cancer prevention, diagnostics and treatment in Uganda in the next decades.

Although some cases diagnosed at autopsy were captured from mortuary departments of the health facilities, the study failed to access the vital statistics data from the birth and death registration office at the National Identification and Regulatory Office (NIRA) due to legislation pertaining to the confidentiality of death certificates. Overall, mortality data in Uganda is patchy, because a significant proportion of deaths and burials occur at home and are rarely reported to municipal councils. In addition, autopsies are not always conducted and specific causes of death are not reported or known.

Although the findings should be interpreted with some caution, this study has several strengths; we built upon epidemiological concepts and existing international guidelines for cancer registration to design methods that could be used to assess cancer incidence in regions without PBCRs. This highlights the importance of developing innovative strategies and alternative surveillance tools tailored to the local context to enhance cancer data in the country. The need for locally-tailored strategies is clear in our work, which, for the first time, presents the best quality population-based cancer data yet produced in these regions. The model we have produced, if replicated, will hopefully result in the production of nationally representative cancer incidence estimates for the country and will ensure that the cancer burden is described according to specific cancer type, geographical location, ethnic origin, age, and sex.

In addition, these results provide further support for the hypothesis that urban PBCRs may not be representative of countries that are largely rural and methods are needed to investigate these differences further. Thus, although not without its complexities, having identified such a high percentage of cases with a resonantly high MV% indicates the success of the study in estimating cancer incidence among the regions and points out a great opportunity for major improvements in cancer care and surveillance systems in Uganda.

## Conclusion

The principal finding is that the overall age-standardised incidence rates observed in this study, all ages and all cancers combined, among males and females, are far below those observed by the Kampala cancer registry 2010–2013 estimates, and the Globacan 2018 estimates for Uganda. This is due to the better capture of non-urban populations in the study sample, compared to the existing registry. The results provided by this study are the first ever incidence rates on a national and regional level for Uganda, derived from a population-based approach. In the absence of any existing population-based cancer data among these regions, this data provides a unique insight into the cancer patterns in these regions. This knowledge improves greatly our understanding of the epidemiology of cancer in Uganda and is an important addition to existing cancer registration data. The model we have produced in this study, covering Central, Western and Eastern regions, could be applied to cover all the regions, providing more accurate estimates of the cancer burden for the whole country. With periodic application, this would allow completion of time trend analysis to truly determine the changing cancer burden in Uganda, and ensure timely disaggregated data for cancer control planning at all levels.

### Electronic supplementary material

Below is the link to the electronic supplementary material.

Supplementary Material 1

## Data Availability

The datasets used and/or analysed during the current study are available from the corresponding author on reasonable request.

## Declarations

### Authors’ information

Dr. Annet Nakaganda is a Clinical Officer and a Principal Cancer Epidemiologist, specialized in the conduct of cancer clinical trials and epidemiological investigations for cancer control in Africa. She is currently the Head of Cancer Epidemiology and Clinical Trials Division at Uganda Cancer Insitute. Dr. Nakaganda holds a PhD in medicine (Cancer Epidemiology) and a Master’s degree in Public Health (Distinction) from the University of Manchester UK. Her recent work is focused on identifying novel approaches for accelerating timely availability and utilization of locally-generated quality cancer data to direct the cancer control effort in low-resource settings.

Annet is trained, and recognized internationally, as a leader in cancer control planning and implementation. She is a supervisor for Field Epidemiology Fellows at the Uganda Cancer Institute and has more than 16 years of cancer research experience, serving in various positions including: principal investigator, research clinician; research coordination; research governance and research monitoring/auditing. Annet has worked to establish the Cancer Epidemiology and Clinical Trials unit at the Uganda Cancer Institute; and has led the harmonization of cancer registration guidelines for East Africa.

Dr. Nakaganda is also trained in higher education curriculum and pedagogy and has extensive experience in teaching at the university level. She began her teaching career in 2016 as a Teaching Associate (TA) at The University of Manchester’s Masters of Public Health Program(MPH) and at the BSc. Biomedical science course, Biochemistry of Disease unit. She supported the design and development of The University of Manchester’s BSc. in Public Health course. She is presently a subject matter expert (SME) and lecturer on the MSc. Clinical Research and MSc. Public Health and Health Promotion curricula at the iHeed-University of Warwick. She also teaches Research Ethics and Good Clinical Practice and has taught over 600 researchers in Research Ethics through around 50 in-country trainings.

She is a winner of several awards including: The HIV Trust Scholarship Award UK; University of Manchester Equity and Merit Scholarship Award; PRIM&R Global research scholarship USA; Union for International Cancer Control (UICC) World Cancer Congress travel grant; University of Mexico Prevent Cancer Foundation Immersion Scholarship Award; University of Manchester Doctoral Academy Research Excellence Award; CUGH-The Lancet Poster Competition Award; and BVGH’s AC3T Study Pool Mini-Grant Award. She has published in peer reviewed local and international journals and conferences.
