# Supplementary material for: Estimating regional and national cancer incidence in Uganda: a retrospective population-based study, 2013–2017
Source: BMC Cancer. 2024 Jul 2;24:787. doi: 10.1186/s12885-024-12543-9 (PMC11218197; doi:10.1186/s12885-024-12543-9)
Supplement: Supplementary file 1 — Supplementary Material 1 [file 12885_2024_12543_MOESM1_ESM.pdf]

Appendix 1: Overall cancer incidence (cases, age-standardized rates, cumulative rates), 2013-2017

**Site**	**Males**				**Females**				
	No. cases	Crude rate Per 100,0000	ASR	Cum. (%) 0-74	No. cases	Crude Rate Per 100,000	ASR	Cum. (%) 0-74	*ICD-10*
Tongue	64	0.5	**1.2**	0.15	15	0.1	**0.3**	0.03	*C01-02*
Mouth	57	0.4	**0.9**	0.10	32	0.2	**0.4**	0.06	*C03-06*
Salivary glands	34	0.3	**0.5**	0.06	29	0.2	**0.3**	0.03	*C07-08*
Nasopharynx	71	0.5	**0.9**	0.10	37	0.3	**0.4**	0.04	*C11*
Hypopharynx	8	0.1	**0.2**	0.03	4	0.0	**0.1**	0.01	*C12-13*
Pharynx unspecified	20	0.1	**0.4**	0.04	12	0.1	**0.2**	0.02	*C14*
Oesophagus	647	4.8	**13.4**	1.63	357	2.5	**6.3**	0.75	*C15*
Stomach	231	1.7	**4.4**	0.50	216	1.5	**3.5**	0.41	*C16*
Colon	138	0.34	**2.7**	0.34	127	0.9	**2.0**	0.24	*C18*
Rectum	108	0.8	**1.9**	0.22	96	0.7	**1.6**	0.20	*C19-20*
Anus	25	0.2	**0.4**	0.05	27	0.2	**0.4**	0.05	*C21*
Liver	256	1.9	**4.1**	0.47	226	1.6	**3.3**	0.36	*C22*
Pancreas	48	0.4	**0.9**	0.11	46	0.3	**0.8**	0.09	*C25*
Nose, sinuses etc.	56	0.4	**0.8**	0.10	32	0.2	**0.4**	0.03	*C30-31*
Larynx	56	0.4	**1.3**	0.18	5	0.0	**0.1**	0.00	*C32*
Trachea, bronchus and lung	110	0.8	**2.0**	0.24	78	0.6	**1.3**	0.16	*C33-34*
Bone	73	0.5	**0.7**	0.05	66	0.5	**0.6**	0.05	*C40-41*
Melanoma of skin	33	0.2	**0.6**	0.06	34	0.2	**0.6**	0.06	*C43*
Other skin	75	0.6	**1.0**	0.11	81	0.6	**1.0**	0.12	*C44*
Kaposi sarcoma	848	6.3	**8.8**	0.79	498	3.5	**3.9**	0.31	*C46*
Connective and soft tissue	75	0.6	**0.8**	0.07	74	0.5	**0.6**	0.05	*C47, 49*
Breast	51	0.4	**0.9**	0.11	1003	7.1	**14.3**	1.53	*C50*
Vulva					76	0.5	**1.1**	0.12	*C51*
Cervix uteri					1802	12.8	**26.8**	2.96	*C53*
Corpus uteri					103	0.7	**1.7**	0.21	*C54*
Uterus unspecified					77	0.5	**1.0**	0.10	*C55*
Ovary					243	1.7	**3.2**	0.35	*C56*
Placenta					20	0.1	**0.1**	0.01	*C58*
Penis	94	0.7	**1.7**	0.17					*C60*
Prostate	878	6.6	**19.4**	2.27					*C61*
Testis	21	0.2	**0.2**	0.01					*C62*
Kidney	66	0.5	**0.6**	0.05	62	0.4	**0.5**	0.04	*C64*
Bladder	56	0.4	**1.1**	0.11	30	0.2	**0.5**	0.05	*C67*
Eye	129	1.0	**1.2**	0.10	169	1.2	**1.3**	0.11	*C69*
Brain, nervous system	41	0.3	**0.4**	0.3	58	0.4	**0.7**	0.07	*C70-72*
Thyroid	18	0.1	**0.3**	0.02	65	0.5	**0.9**	0.10	*C73*
Hodgkin disease	122	0.8	**1.0**	0.08	86	0.6	**0.8**	0.07	*C81*
Non-Hodgkin lymphoma	258	1.9	**2.7**	0.27	191	1.4	**1.9**	0.18	*C82-85, C96*
Multiple myeloma	50	0.4	**1.0**	0.12	51	0.4	**0.9**	0.11	*C90*
Lymphoid leukaemia	19	0.1	**0.2**	0.03	7	0.0	**0.1**	0.01	*C91*
Myeloid leukaemia	75	0.6	**0.8**	0.06	55	0.4	**0.5**	0.04	*C92-94*
Leukaemia unspecified	73	0.5	**0.6**	0.06	45	0.3	**0.3**	0.02	*C95*
Other and unspecified	125	0.9	**2.0**	0.21	132	0.9	**1.7**	0.17	*O&U*
All sites	5155	38.6	**82.9**	9.21	6436	45.8	**87.4**	9.45	*All*
*All sites but C44	5080	38.0	**81.9**	9.10	6355	45.2	**86.4**	9.33	*AllbC44*

*Table produced by CanReg5; ASR=Age-standardised incidence rate; Cum.= Cumulative rate; * Non-melanoma skin cancer (C44) is often excluded from comparative analyses of cancer data, because of concerns about the completeness of registration (case ascertainment) and a perception that cancers of this kind are rarely life threatening.*

Appendix 2: Cancer incidence (cases, age-standardized rates, cumulative rates), central region, 2013-2017

Site	Males				Females				
	No. cases	Crude rate Per 100,0000	ASR	Cum. (%) 0-74	No. cases	Crude Rate Per 100,000	ASR	Cum. (%) 0-74	*ICD-10*
Tongue	49	0.8	**2.1**	0.26	12	0.2	**0.5**	0.06	*C01-02*
Mouth	39	0.6	**1.4**	0.16	22	0.3	**0.8**	0.11	*C03-06*
Salivary glands	20	0.3	**0.6**	0.07	20	0.3	**0.5**	0.04	*C07-08*
Nasopharynx	59	0.9	**1.8**	0.20	27	0.4	**0.7**	0.06	*C11*
Pharynx unspecified	10	0.2	**0.4**	0.07	6	0.1	**0.2**	0.03	*C14*
Oesophagus	340	5.4	**17.4**	2.20	214	3.2	**9.2**	1.07	*C15*
Stomach	125	2.0	**5.9**	0.68	104	1.5	**4.4**	0.53	*C16*
Colon	94	1.5	**4.3**	0.60	90	1.3	**3.6**	0.44	*C18*
Rectum	87	1.4	**3.5**	0.41	68	1.0	**2.8**	0.38	*C19-20*
Anus	15	0.2	**0.7**	0.09	22	0.3	**0.8**	0.09	*C21*
Liver	164	2.6	**6.0**	0.73	145	2.1	**5.1**	0.58	*C22*
Gallbladder etc.	2	0.0	**0.0**	0.00	12	0.2	**0.5**	0.05	*C22-24*
Pancreas	29	0.5	**1.3**	0.16	31	0.5	**1.4**	0.16	*C25*
Nose, sinuses etc.	39	0.6	**1.5**	0.19	20	0.3	**0.6**	0.06	*C30-31*
Larynx	39	0.6	**2.3**	0.33	4	0.1	**0.1**	0.00	*C32*
Trachea, bronchus and lung	80	1.3	**3.6**	0.47	57	0.8	**2.3**	0.28	*C33-34*
Bone	50	0.8	**1.0**	0.07	35	0.5	**0.8**	0.07	*C40-41*
Melanoma of skin	10	0.2	**0.4**	0.05	14	0.2	**0.6**	0.06	*C43*
Other skin	56	0.9	**1.7**	0.22	56	0.8	**1.6**	0.19	*C44*
Kaposi sarcoma	682	10.7	**14.1**	1.29	410	6.1	**6.5**	0.52	*C46*
Connective and soft tissue	53	0.8	**1.2**	0.10	54	0.8	**1.1**	0.09	*C47, 49*
Breast	37	0.6	**1.7**	0.18	737	10.9	**25.0**	2.77	*C50*
Vulva					54	0.8	**1.9**	0.22	*C51*
Cervix uteri					1074	15.9	**37.3**	4.17	*C53*
Corpus uteri					72	1.1	**3.4**	0.41	*C54*
Uterus unspecified					50	0.7	**1.3**	0.13	*C55*
Ovary					153	2.3	**4.7**	0.54	*C56*
Placenta					18	0.3	**0.3**	0.02	*C58*
Penis	60	0.9	**2.3**	0.23					*C60*
Prostate	512	8.1	**29.8**	3.70					*C61*
Testis	11	0.2	**0.2**	0.02					*C62*
Kidney	45	0.7	**1.1**	0.10	45	0.7	**0.8**	0.07	*C64*
Bladder	40	0.6	**1.9**	0.20	22	0.3	**1.0**	0.10	*C67*
Eye	71	1.1	**1.5**	0.12	102	1.5	**1.8**	0.15	*C69*
Brain, nervous system	27	0.4	**0.6**	0.05	39	0.6	**1.1**	0.13	*C70-72*
Thyroid	15	0.2	**0.5**	0.03	50	0.7	**1.6**	0.19	*C73*
Hodgkin disease	68	1.1	**1.3**	0.10	58	0.9	**1.2**	0.11	*C81*
Non-Hodgkin lymphoma	177	2.8	**4.2**	0.42	120	1.8	**2.9**	0.29	*C82-85, C96*
Multiple myeloma	38	0.6	**1.9**	0.22	34	0.5	**1.5**	0.20	*C90*
Lymphoid leukaemia	17	0.3	**0.4**	0.04	5	0.1	**0.2**	0.03	*C91*
Myeloid leukaemia	50	0.8	**1.1**	0.08	38	0.6	**0.9**	0.07	*C92-94*
Leukaemia unspecified	47	0.7	**0.9**	0.08	28	0.4	**0.5**	0.04	*C95*
Other and unspecified	80	1.3	**3.0**	0.32	81	1.2	**2.5**	0.27	*O&U*
All sites	3383	53.2	**125.4**	14.50	4236	62.8	**134.6**	14.86	*All*
*All sites but C44	3327	52.4	**123.7**	14.28	4180	61.9	**133.0**	14.67	*AllbC44*

*Table produced by CanReg5; ASR=Age-standardised incidence rate; Cum.= Cumulative rate; * Non-melanoma skin cancer (C44) is often excluded from comparative analyses of cancer data, because of concerns about the completeness of registration (case ascertainment) and a perception that cancers of this kind are rarely life threatening*

Appendix 3: Cancer incidence (cases, age-standardized rates, cumulative rates), Western region 2013-2017

Site	Males				Females				
	No. cases	Crude rate Per 100,0000	ASR	Cum. (%) 0-74	No. cases	Crude Rate Per 100,000	ASR	Cum. (%) 0-74	*ICD-10*
Tongue	12	0.3	**0.6**	0.08	2	0.1	**0.1**	0.02	*C01-02*
Mouth	14	0.4	**0.7**	0.09	6	0.2	**0.2**	0.03	*C03-06*
Salivary glands	6	0.2	**0.3**	0.02	4	0.1	**0.1**	0.02	*C07-08*
Nasopharynx	5	0.1	**0.2**	0.03	7	0.2	**0.3**	0.04	*C11*
Pharynx unspecified	6	0.2	**0.4**	0.05	1	0.0	**0.1**	0.00	*C14*
Oesophagus	177	4.6	**11.0**	1.27	49	1.2	**2.6**	0.37	*C15*
Stomach	92	2.4	**5.5**	0.61	74	1.9	**3.7**	0.42	*C16*
Colon	23	0.6	**1.4**	0.15	21	0.5	**1.1**	0.12	*C18*
Rectum	13	0.3	**0.9**	0.13	20	0.5	**1.1**	0.13	*C19-20*
Anus	8	0.2	**0.4**	0.04	2	0.1	**0.1**	0.02	*C21*
Liver	60	1.6	**3.4**	0.38	46	1.2	**2.1**	0.23	*C22*
Gallbladder	1	0.0	**0.1**	0.01	5	0.1	**0.3**	0.05	*C23-24*
Pancreas	13	0.3	**0.8**	0.09	11	0.3	**0.5**	0.05	*C25*
Nose, sinuses etc.	10	0.3	**0.5**	0.06	10	0.3	**0.3**	0.03	*C30-31*
Larynx	15	0.4	**1.1**	0.13	0	0.0	**0.0**	0.00	*C32*
Trachea, bronchus and lung	22	0.6	**1.3**	0.12	17	0.4	**0.9**	0.11	*C33-34*
Bone	10	0.3	**0.4**	0.03	13	0.3	**0.3**	0.02	*C40-41*
Melanoma of skin	8	0.2	**0.6**	0.07	12	0.3	**0.6**	0.06	*C43*
Other skin	13	0.3	**0.6**	0.06	10	0.3	**0.4**	0.04	*C444*
Kaposi sarcoma	127	3.3	**4.8**	0.42	55	1.4	**1.5**	0.11	*C46*
Connective and soft tissue	16	0.4	**0.5**	0.03	9	0.2	**0.2**	0.02	*C47,C49*
Breast	9	0.2	**0.5**	0.08	147	3.7	**6.8**	0.71	*C50*
Vulva					15	0.4	**0.7**	0.07	*C51*
Cervix uteri					415	10.5	**20.3**	2.33	*C53*
Corpus uteri					14	0.4	**0.7**	0.07	*C54*
Uterus unspecified					15	0.4	**0.8**	0.09	*C55*
Ovary					54	1.4	**2.2**	0.22	*C56*
Placenta					2	0.1	**0.0**	0.00	*C58*
Penis	23	0.6	**1.3**	0.13					*C60*
Prostate	204	5.3	**12.8**	1.38					*C61*
Testis	9	0.2	**0.3**	0.02					*C62*
Kidney	9	0.2	**0.2**	0.01	9	0.2	**0.2**	0.03	*C64*
Bladder	4	0.1	**0.3**	0.05	5	0.1	**0.2**	0.02	*C67*
Eye	39	1.0	**1.2**	0.10	42	1.1	**1.1**	0.09	*C69*
Brain, nervous system	7	0.2	**0.3**	0.04	14	0.4	**0.6**	0.05	*C70-71*
Thyroid	0	0.0	**0.0**	0.00	9	0.2	**0.3**	0.04	*C73*
Hodgkin disease	33	0.9	**1.1**	0.09	15	0.4	**0.5**	0.05	*C81*
Non-Hodgkin lymphoma	50	1.3	**1.8**	0.17	42	1.1	**1.2**	0.10	*C82-85,C96*
Multiple myeloma	6	0.2	**0.4**	0.04	12	0.3	**0.7**	0.08	*C90*
Lymphoid leukaemia	2	0.1	**0.1**	0.03	2	0.1	**0.0**	0.00	*C91*
Myeloid leukaemia	20	0.5	**0.7**	0.06	13	0.3	**0.4**	0.04	*C92-94*
Leukaemia unspecified	12	0.3	**0.3**	0.02	11	0.3	**0.2**	0.01	*C95*
Other and unspecified	32	0.8	**1.7**	0.20	32	0.8	**1.4**	0.13	*O&U*
**All sites**	1117	29.2	**58.8**	6.32	1256	31.9	**55.7**	6.12	*ALL*
***All sites but C44**	1104	28.9	**58.2**	6.26	1247	31.7	**55.3**	6.09	*ALLbC44*

*Table produced by CanReg5; ASR=Age-standardised rate; Cum.=Cumulative rate; * Non-melanoma skin cancer (C44) is often excluded from comparative analyses of cancer data, because of concerns about the completeness of registration (case ascertainment) and a perception that cancers of this kind are rarely life threatening.*

Appendix 4: Cancer incidence (cases, age-standardized rates, cumulative rates) Eastern region, 2013-2017

Site	Males				Females				
	No. cases	Crude rate Per 100,0000	ASR	Cum. (%) 0-74	No. cases	Crude Rate Per 100,000	ASR	Cum. (%) 0-74	*ICD-10*
Tongue	3	0.1	**0.3**	0.06	1	0.0	**0.1**	0.01	*C01-02*
Mouth	4	0.1	**0.2**	0.03	4	0.1	**0.2**	0.03	*C03-06*
Salivary glands	8	0.3	**0.6**	0.08	5	0.1	**0.3**	0.03	*C07-08*
Nasopharynx	7	0.2	**0.3**	0.01	3	0.1	**0.2**	0.01	*C11*
Pharynx unspecified	4	0.1	**0.3**	0.02	5	0.1	**0.3**	0.03	*C14*
Oesophagus	130	4.1	**10.7**	1.28	94	2.8	**6.6**	0.75	*C15*
Stomach	14	0.4	**1.0**	0.13	38	1.1	**2.3**	0.26	*C16*
Colon	21	0.7	**1.7**	0.20	16	0.5	**0.9**	0.09	*C18*
Rectum	8	0.3	**0.5**	0.06	8	0.2	**0.5**	0.06	*C19-20*
Anus	2	0.1	**0.1**	0.01	3	0.1	**0.2**	0.03	*C21*
Liver	32	1.0	**2.0**	0.18	35	1.0	**2.1**	0.22	*C22*
Pancreas	6	0.2	**0.5**	0.06	4	0.1	**0.2**	0.04	*C25*
Nose, sinuses etc.	7	0.2	**0.4**	0.02	2	0.1	**0.1**	0.01	*C30-31*
Larynx	5	0.2	**0.3**	0.04	1	0.0	**0.1**	0.01	*C32*
Trachea, bronchus and lung	8	0.3	**0.5**	0.05	4	0.1	**0.3**	0.03	*C33-34*
Bone	13	0.4	**0.6**	0.05	18	0.5	**0.6**	0.04	*C40-41*
Melanoma of skin	15	0.5	**0.9**	0.07	8	0.2	**0.6**	0.08	*C43*
Other skin	6	0.2	**0.3**	0.02	16	0.5	**0.8**	0.10	*C444*
Kaposi's sarcoma	39	1.2	**2.3**	0.23	33	1.0	**1.5**	0.13	*C46*
Connective and soft tissue	6	0.2	**0.3**	0.05	11	0.3	**0.4**	0.03	*C47,C49*
Breast	5	0.2	**0.4**	0.04	119	3.5	**6.8**	0.70	*C50*
Vulva					7	0.2	**0.4**	0.04	*C51*
Cervix uteri					313	9.3	**18.4**	1.93	*C53*
Corpus uteri					17	0.5	**1.0**	0.12	*C54*
Uterus unspecified					12	0.4	**0.7**	0.07	*C55*
Ovary					36	1.1	**2.0**	0.21	*C56*
Penis	11	0.3	**1.0**	0.12					*C60*
Prostate	162	5.1	**14.2**	1.42					*C61*
Testis	1	0.0	**0.0**	0.00					*C62*
Kidney	12	0.4	**0.4**	0.02	8	0.2	**0.3**	0.03	*C64*
Bladder	12	0.4	**1.0**	0.05	3	0.1	**0.2**	0.01	*C67*
Eye	19	0.6	**0.8**	0.06	25	0.7	**0.8**	0.06	*C69*
Brain, nervous system	7	0.2	**0.3**	0.02	5	0.1	**0.2**	0.01	*C70-71*
Thyroid	3	0.1	**0.2**	0.02	6	0.2	**0.4**	0.04	*C73*
Hodgkin disease	11	0.3	**0.4**	0.02	13	0.4	**0.5**	0.03	*C81*
Non-Hodgkin lymphoma	31	1.0	**1.2**	0.13	29	0.9	**1.1**	0.10	*C82-85,C96*
Multiple myeloma	6	0.2	**0.5**	0.07	5	0.1	**0.3**	0.04	*C90*
Myeloid leukaemia	5	0.2	**0.3**	0.04	4	0.1	**0.2**	0.01	*C92-94*
Leukaemia unspecified	14	0.4	**0.6**	0.06	6	0.2	**0.2**	0.01	*C95*
Other and unspecified	12	0.4	**0.6**	0.06	19	0.6	**0.9**	0.08	*O&U*
**All sites**	655	20.5	**45.8**	4.83	944	28.1	**53.1**	5.52	*ALL*
***All sites but C44**	649	20.3	**45.5**	4.81	928	27.6	**52.3**	5.41	*ALLbC44*

*Table produced by CanReg5; ASR=Age standardise incidence rate; Cum. =Cumulative rate; * Non-melanoma skin cancer (C44) is often excluded from comparative analyses of cancer data, because of concerns about the completeness of registration (case ascertainment) and a perception that cancers of this kind are rarely life threatening.*
